# Teaching and learning in clinical placements: associations of supervision and learning opportunities with placement experience in NETS 2025

**DOI:** 10.3389/fmed.2026.1947428

**Published:** 2026-10-28

**Authors:** Liang Liu, Wanying Zhang, Qingfei Chu

**Affiliations:** 1 Department of Orthopedic Surgery, The Second Affiliated Hospital, Zhejiang University School of Medicine, Hangzhou, China; 2 Department of Infectious Diseases, The Second Affiliated Hospital, Zhejiang University School of Medicine, Hangzhou, China

**Keywords:** clinical supervision, learning affordances, NETs, organization-level analysis, placement experience, workplace learning

## Abstract

**Background:**

Clinical supervision and access to learning opportunities are associated dimensions of the clinical learning environment. Their associations with perceived placement experience are relevant to organizational review of educational quality. This study examined associations among three National Education and Training Survey (NETS) 2025 indicators using a workplace learning affordances perspective.

**Methods:**

We analyzed public organization-level aggregate scores from the NETS 2025 reporting tool. An extraction audit during revision corrected incomplete pagination and compressed-value decoding errors in the initial extract. The corrected dataset included 743 reporting organizations with complete Supervision, Teaching & Learning, and Overall Experience scores. Ordinary least squares models used HC3 robust standard errors. A theory-informed statistical decomposition estimated an indirect association, with percentile confidence intervals from 10,000 organization-level bootstrap resamples. Sensitivity analyses examined boundary scores, influential observations, additional indicators, respondent-count weighting, minimum respondent counts, and clustering.

**Results:**

Supervision was positively associated with Overall Experience (B = 0.838, 95% CI 0.794–0.883) and Teaching & Learning (B = 0.789, 95% CI 0.742–0.835). Teaching & Learning remained associated with Overall Experience after adjustment for Supervision (B = 0.375, 95% CI 0.306–0.443). The indirect association was 0.295 (bootstrap 95% CI 0.240–0.351), or 0.314 after standardization (95% CI 0.258–0.367). The outcome model had R^2^ = 0.837 and adjusted R^2^ = 0.836. Sensitivity estimates remained positive but varied in magnitude.

**Conclusions:**

Organizations with higher supervision scores also had higher perceived teaching and learning quality and overall experience scores. The cross-sectional decomposition identifies a positive indirect component whose magnitude varies across specifications. These findings support reviewing supervision, access to learning opportunities, and overall experience as a connected profile when interpreting organizational survey feedback. These organization-level associations do not establish individual-level effects or causal mediation.

## 1 Introduction

Clinical placements connect the formal curriculum with the everyday work of healthcare. Learners encounter patients, observe professional judgement, participate in teams, and develop their understanding of what competent practice involves. The educational value of these experiences depends on the interaction between opportunities to participate and the guidance available during participation. Experience-based learning accounts describe supported involvement in authentic clinical work as central to professional development, with relationships, emotions, and practical responsibilities contributing to the experience (1, 2). Clinical supervision gives these experiences structure through observation, feedback, discussion, and review of progress (3, 4). Understanding how learners experience supervision alongside their wider learning opportunities is therefore relevant to both placement design and educational quality assurance.

The clinical learning environment encompasses personal, social, organizational, and material dimensions. Gruppen and colleagues describe these dimensions as overlapping and interactive, while Nordquist and colleagues identify architectural, digital, educational, psychological, sociocultural, and inclusion-related perspectives on the same environment (5, 6). A concept analysis in nursing similarly identifies physical space, psychosocial interaction, organizational culture, and teaching and learning as defining attributes (7). These frameworks direct attention to the arrangements through which everyday clinical work becomes accessible to learners: who welcomes them, which tasks they can undertake, how feedback is provided, and whether educational activities fit within service demands. Together, these arrangements shape an environment that learners experience as a connected whole.

Recent placement research provides concrete descriptions of these connections. Cant and colleagues examined 3,640 free-text comments from a national Australian nursing placement survey. Students linked support with a welcoming environment, learning opportunities, and teaching strategies (8). In an English NHS evaluation of a student-led clinical learning environment, Channa and colleagues found that support from peers, clinical educators, and ward staff featured prominently in students’ accounts of confidence and professional development (9). These studies position supportive relationships and access to meaningful activity alongside each other. They also suggest that survey indicators of supervision and educational opportunity can provide complementary information about the same placement experience.

Quantitative studies have examined related patterns across professions and settings. A Moroccan survey of 1,175 nursing and midwifery students identified differences in satisfaction across clinical learning environment dimensions, with supervisory relationships and opportunities for consistent supervisor meetings receiving particular attention (10). A mixed-methods study across health professional programmes in Qatar connected perceptions of the learning environment with professional identity and identified mentorship and emotional support as features of students’ accounts (11). Among nursing students in Iran, perceptions of the clinical education environment were associated with academic adjustment (12). More recently, a national survey of respiratory therapy students in Saudi Arabia described variation in learning environment ratings across academic and clinical contexts (13). This work establishes a broad empirical interest in how relational support, learning arrangements, and placement appraisals are connected.

The organization of supervision itself is diverse. Zonneveld and colleagues identified 33 models and frameworks in a scoping review of nursing supervision, illustrating the range of purposes, structures, and settings in which the term is used (14). Gill-Meeley and colleagues synthesized nurses’ and midwives’ experiences across 15 countries, including individual and group supervision (15). Awiagah and colleagues highlighted collaboration, feedback, training, and implementation conditions in their review of supervision in Ghana (16). For placement evaluation, these findings support attention to the supervisory activities learners actually encounter. Timely feedback and progress review provide observable points of reference within this broader field of supervisory practice.

Measurement research examines how item content, dimensionality, and intended populations shape the interpretation of clinical learning environment scores (17, 18). Recent work has developed a context-specific scale for operating-room placements and examined the measurement of feedback literacy in health professions students (19, 20). The choice of survey items therefore helps determine which aspects of experience a score represents. A useful analysis should connect statistical relationships with the educational content of the corresponding indicators, allowing readers to understand what a high or low value means in the setting under study.

The National Education and Training Survey (NETS) provides a public source of organization-level information about healthcare learners’ practice placements and training posts. Its reporting tool includes scores for Supervision, Teaching & Learning, Overall Experience, and other dimensions of the placement environment (21). Public organization-level reporting offers a perspective relevant to providers reviewing educational quality across services. It enables examination of whether organizations with favorable supervision ratings also report favorable learning opportunities and overall experiences, and whether the associations persist when reporting size and administrative context are considered. The reporting organization is the unit around which the present analysis and its practical interpretation are organized.

Existing empirical studies have mainly examined individual learners, specific programmes, or particular placement models. Their findings motivate a further question about the joint pattern of these three published NETS indicators across reporting organizations. The present study estimates the association between Teaching & Learning and Overall Experience conditional on Supervision, quantifies the indirect component in the Supervision–Overall Experience relationship, and examines how that component varies across analytical specifications. This brings the content of the indicators, their observed covariance, and the robustness of the estimated relationships into a common analysis. The resulting evidence can help educational teams interpret combinations of survey scores when deciding which aspects of placement experience warrant closer local examination.

### 1.1 Workplace learning affordances

We use workplace learning affordances to describe opportunities and invitations to participate in work-related activities and interactions, including access to guidance and feedback. In Billett's account, learning through work involves both the opportunities offered by the workplace and the ways individuals engage with them (22, 23). A pedagogically rich workplace offers accessible participation, guidance, and feedback relevant to learners’ development. Its educational character is expressed in everyday practices, such as inviting a learner to discuss a patient, explaining a clinical decision, making time for supervised participation, or helping the learner revisit an encounter after receiving feedback.

This perspective connects the relational and organizational aspects of placement learning. A scheduled learning opportunity becomes educationally useful through participation: the learner needs access to the activity, an appropriate role, and guidance suited to the task. Supervisory relationships provide one means of negotiating these conditions. They may help learners understand expectations, ask for assistance, identify suitable responsibilities, and obtain feedback on performance. The individual learner contributes prior knowledge, confidence, interests, and willingness to engage. Across a placement, opportunities and engagement are repeatedly renegotiated as the learner becomes more familiar with the work and with the clinical team.

The educational alliance provides a related account of how supervisory relationships support feedback. Telio and colleagues conceptualize feedback within a negotiated educational relationship involving shared goals, engagement, and mutual understanding (24). Their subsequent study describes how residents’ judgements of a supervisor's credibility and commitment to learning influence their engagement with feedback (25). Watling and Ginsburg further emphasize the influence of assessment culture on how feedback is interpreted and used (26). These perspectives make the perceived quality of supervision educationally relevant: the learner's appraisal includes the availability, credibility, and usefulness of support within ongoing clinical activity.

Psychological safety also helps describe the relational conditions of participation. Bump and Cladis discuss its relevance to admitting uncertainty and engaging in learning, while Dong and colleagues consider setting-specific strategies across health professions education (27, 28). In a qualitative study of online group supervision for student mental health nurses, Howard and Peirson identified confidence, trust, and familiarity as important features of participation (29). These accounts suggest that opportunities are experienced through relationships as well as through timetables and resources. In the present study, the affordances perspective organizes the educational interpretation of supervision, access to learning, and overall placement appraisal. Workplace learning denotes this broad setting and process; experience-based learning refers to the particular theoretical accounts cited above.

### 1.2 Indicator distinctions and analytical expectations

The three NETS indicators describe associated, partly overlapping dimensions of placement experience. Supervision includes perceived supervisory quality, timely and constructive feedback, and review of progress. Teaching & Learning covers the range of opportunities, permission to attend relevant activities, learning with other professions, and access to simulation. Overall Experience captures a broader appraisal, including the educational experience, the value placed on education, supportive staff, and recommendations concerning care and training. Their question content and regression roles are summarized in Table 1. This item-based interpretation allows the analysis to connect general theoretical concepts with specific aspects of the public reporting system.

**Table 1:** NETS indicators and conceptual boundaries.

Indicator and role	Current mapped questions	Content summary
Supervision (X)	Q20.1, Q20.2, Q20.3, Q20.5	Overall supervision; timeliness and usefulness of feedback; review of progress. Includes ongoing guidance and feedback.
Teaching & Learning (M)	Q19.3, Q19.4, Q19.5, Q22.5	Range of opportunities; attendance at required learning activities; learning with other professions; simulation access. Measures perceived access to educational opportunities.
Overall Experience (Y)	Q19.2, Q20.7, Q22.7, Q31.1, Q31.2	Valuing education; overall educational experience; welcoming and supportive staff; recommendations for care and for work or training. A broad perceived placement appraisal.

Source: current NETS 2025 public question mapping and scoring metadata. Question labels are paraphrased. M is the intermediate variable in the cross-sectional statistical decomposition. All three published scores use a normalized 0–100 scale; conceptual overlap remains possible. Item summaries appear in Supplementary Table S2; verbatim question wording and response options appear in Supplementary Tables S9A–S9C.

Supervision is expected to be positively associated with overall experience because guidance and support form part of the learner's appraisal of a placement. The literature on supervision and educational alliances provides a rationale for this association through the perceived availability and usefulness of feedback, clarity of expectations, and continuity of support (4, 24). Supervision is also expected to be positively associated with Teaching & Learning because clinical guidance and access to educational activities occur within the same working arrangements. The student narratives reported by Cant and colleagues and the English placement evaluation by Channa and colleagues illustrate this conjunction of staff support and opportunities to participate (8, 9).

Teaching & Learning is expected to retain an association with Overall Experience after accounting for Supervision because opportunities to undertake relevant activities contribute additional information about the placement. A learner can evaluate both how support is provided and what educational activity is available. The NETS decomposition estimates how these two dimensions relate jointly to the overall appraisal. The product of the Supervision–Teaching & Learning coefficient and the conditional Teaching & Learning–Overall Experience coefficient provides a summary of the indirect component under the specified ordering.

Accordingly, four associational expectations guide the analysis: H1, Supervision is positively associated with Overall Experience; H2, Supervision is positively associated with Teaching & Learning; H3, Teaching & Learning is positively associated with Overall Experience conditional on Supervision; and H4, the product of the relevant regression coefficients is positive. The primary estimand is the between-organization association under equal organization weighting. Sensitivity analyses examine its stability across score boundaries, influential observations, additional placement indicators, reporting-size specifications, and administrative clustering. This sequence links a clearly specified aggregate research question to both the theoretical framing and the practical use of the NETS reporting system.

## 2 Materials and methods

### 2.1 Design and reporting unit

This was a cross-sectional secondary analysis of public NETS 2025 aggregate scores. A reporting organization, identified by its published organization name, was the unit of analysis. These heterogeneous units include trusts, primary care networks or general-practice organizations, and other providers. The public extract covers healthcare learners from different professional and training groups. The estimand is an association between organization-level scores.

NETS concerns healthcare training in England. We retained the complete set produced by the specified reporting query and describe the population as NETS reporting organizations. The contextual audit identified six organizations with administrative labels outside England, together with missing or multiple administrative mappings. The retrieved population therefore follows the reporting view and its filters. Available provider categories and administrative labels were used to describe this population and define the clustering sensitivity subset.

### 2.2 Extraction audit and dataset construction

The original extraction was made on 7 July 2026. During revision, we audited the retained Power BI query payloads and responses against the analysis file. Two extraction errors were identified. First, a matrix response stopped at 500 organizations and included a continuation token, indicating incomplete retrieval. Second, compressed repeated-value fields had been decoded incorrectly, introducing erroneous missing values and numeric values. The revised analyses use the corrected extraction throughout.

On 20 September 2026, we retrieved a complete flat organization-by-indicator table from the same public report, preserving the original query filters and the official masked score measure. The report model recorded the same refresh date, 23 April 2026, in the retained and newly retrieved metadata. The reporting-view filters required HEI_Exclude=false, excluded organization codes beginning with EE, and excluded the named organization list in the retained query. That exact list and query are retained in the authors’ extraction records. The query retained the report's coverage across professions and training stages.

The complete response contained 743 organization names and an explicit completion flag, without a continuation token. The 13 indicators used in the original extract were retained and pivoted by organization name. We independently decoded the archived matrix response, including repeated and null-value bitmasks, and compared all 6,500 archived organization–indicator cells with the new table; every correctly decoded value agreed within numerical tolerance. The original CSV differed in 426 cells. Supplementary Methods S1 and Supplementary Table S1 document the audit and provide examples. The retrieved records are public organization-level aggregates. Published score values were analyzed directly on their original scale.

### 2.3 Measures and score interpretation

The variable X was Supervision, the intermediate variable M was Teaching & Learning, and the outcome variable Y was Overall Experience. These labels specify the variables’ roles in the cross-sectional regression decomposition. We used the official organization-level score measure, ind Mean 25 MASKED. The current report's question mapping and scoring metadata were inspected to identify relevant item content and response coding. Table 1 summarizes the measures, and Supplementary Tables S9A–S9C reproduce the 13 mapped question texts, scored response options, and unscored response categories. The 2025 mapping retains some column names containing 2024; current QCode25 identifiers were matched to the current scoring table to establish the relevant item definitions.

The response categories are normalized to 0, 25, 50, 75, and 100, with higher values indicating more favorable evaluations; non-applicable responses are unscored. The current report's scoring metadata define the normalized response scale (21); the historical HEE guidance for the 2019 survey provides background on the reporting approach (30). We used the published aggregate scores directly. A score of 75 represents a position on this normalized rating scale. A genuine zero would denote the least favorable end of the scale; the apparent zeros in the original three-indicator file arose from decoding errors. The corrected dataset contained positive values for all three primary indicators.

The mapped content differentiates supervision and feedback, access to learning opportunities, and a broader appraisal of the placement environment. The educational content of the indicators overlaps, and all three draw on evaluations within the same survey. The 13 mapped questions have distinct identifiers across the three indicators (Table 1); the concern is conceptual overlap rather than reuse of the same mapped item. For example, the Overall Experience items about supportive staff and the overall educational experience are conceptually close to supervision and access to learning opportunities. Our interpretation therefore concerns overlapping dimensions of perceived placement experience. Instrument research provides the broader measurement context (17, 18), while the current item mapping defines the specific content analyzed here. Measurement-validation questions are addressed in Section 4.6.

### 2.4 Missingness, respondent counts and organizational context

All 743 reporting organizations had observed values on the three primary indicators and on Induction, Facilities, and Workload, allowing the corrected analysis to include every retrieved organization. The previously reported 28 exclusions resulted from decoding errors. The data-completeness assessment therefore concerns the fully retrieved reporting table; public masking and survey participation determine which organizations enter that table. Supplementary Table S3 reports completeness and the distribution of displayed respondent counts.

The report states that results for fewer than three respondents are masked. Public masked organization-level respondent counts were retrievable and ranged from 3 to 1,357, with a median of 6, for each primary indicator. We used the displayed counts as a measure of reporting size. Primary models gave each organization equal weight, estimating the association across reporting organizations. Additional analyses gave greater emphasis to larger reporting groups and examined a minimum-count subset.

We also retrieved public aggregate administrative context. Organization names could map to more than one provider category or integrated care board (ICB). We assigned a single category when the available non-missing labels agreed and otherwise marked the mapping as multiple. Provider composition is reported in Supplementary Table S8. Cluster-sensitive inference was examined in the subset with an unambiguous NHS ICB assignment, thereby linking that sensitivity analysis to a reproducible administrative grouping.

### 2.5 Statistical analysis

Analyses used R 4.6.0. All estimates were obtained by executing R scripts developed for this analysis. Descriptive statistics included means, standard deviations, ranges, medians, and interquartile ranges. Pearson and Spearman correlations characterized pairwise associations. We fitted three ordinary least squares equations: Y on X, M on X, and Y on X and M. Their coefficients are denoted c, a, b, and c′ in the usual path notation. HC3 robust standard errors, t-based confidence intervals, and two-sided *p* values were calculated. The HC3 implementation was cross-checked against an independent matrix calculation. We report conventional and adjusted R^2^ and standardized coefficients. Inferential tests are exploratory; *p* values are reported without multiplicity adjustment.

The product ab summarizes the indirect association under the chosen linear decomposition; c = c′ + ab in the unadjusted linear model. We used 10,000 non-parametric bootstrap resamples of whole organizations, with seed 20260707, to obtain percentile 95% confidence intervals. The standardized product equals ab multiplied by SD(X)/SD(Y); its bootstrap interval recalculates this scaling within each resample. The use of a resampling interval follows methodological work on inference for regression-coefficient products (31, 32). The reported product describes the indirect component of contemporaneous organization-level associations under the specified equations.

Diagnostics included scatterplots with linear and locally smoothed fits, residual-versus-fitted plots, normal quantile plots, leverage, studentized residuals, and Cook's distance. Exploratory HC3 Wald tests examined a quadratic term in the intermediate-variable model and joint quadratic terms in the outcome model. A further descriptive sensitivity analysis allowed a quadratic X–M association. It estimated changes in the fitted M score for a 10-point X contrast at selected observed percentiles, multiplied by the conditional M–Y coefficient. These contrasts describe how the fitted indirect component varies across the observed score range.

### 2.6 Sensitivity analyses

We repeated the decomposition after excluding any organization with a score of 0 or 100 on a primary indicator, and separately after excluding cases with Cook's distance greater than 4/743 in the primary outcome model. A third model included Induction, Facilities, and Workload in all three equations. The original manuscript documented their availability and their perceived lower conceptual redundancy with Teaching & Learning than Teamwork or Wellbeing as the selection rationale. The retained analysis script contains the same three-variable specification. We retained these variables for comparability and report this model as an exploratory conditional-association analysis. The uncertainty about selection timing and the interpretation of adjustment are discussed in Section 4.6.

To examine differences in reporting size, we fitted weighted models using the minimum displayed count across the three indicators as a common organization weight, and separately restricted the sample to organizations with counts of at least 10. The weights emphasize organizations with larger displayed reporting groups. Standardized weighted coefficients were scaled using the unweighted organization-level standard deviations to retain a common reporting convention.

We additionally repeated the analysis in the correctly decoded original set of 500 organizations, separating correction of numeric values from recovery of the remaining organizations. Finally, the 676 organizations with an unambiguous NHS ICB assignment were analyzed with CR2 cluster-robust standard errors and Satterthwaite degrees of freedom across 42 ICBs (33, 34). An ICB-level bootstrap sampled whole clusters with replacement 10,000 times for the indirect association. Table 5 summarizes all specifications, with full paths and inference in Supplementary Tables S4A–S4G, S5.

## 3 Results

### 3.1 Reporting organizations and score distributions

The corrected analytic sample comprised 743 organizations. The available provider labels included 287 general-practice organizations, 178 trusts, 92 independent providers, 57 dental practices, and 35 pharmacy providers; 42 organizations had multiple provider labels and 23 lacked a usable label. Other categories accounted for the remaining organizations. These labels describe the public report's administrative context.

Mean scores were 77.84 for Supervision, 73.33 for Teaching & Learning, and 81.24 for Overall Experience (Table 2). Medians and interquartile ranges also showed predominantly favorable ratings, with variation across organizations. Twenty-four organizations had at least one score of 100; none had a zero on a primary indicator. The overall pattern indicates favorable reported placement experiences among the visible organizations.

**Table 2:** Descriptive statistics across 743 reporting organizations.

Indicator	Mean (SD)	Median [IQR]	Range
Supervision	77.84 (10.92)	77.34 [71.24, 85.42]	25.00–100.00
Teaching & Learning	73.33 (10.36)	72.99 [67.38, 80.56]	20.83–100.00
Overall Experience	81.24 (10.28)	81.25 [75.00, 88.36]	13.33–100.00

*N* = 743 for every indicator. Scores are normalized aggregate ratings on a 0–100 scale. IQR, interquartile range. All primary-indicator values are observed and positive after correcting the extraction.

### 3.2 Pairwise and conditional associations

Pearson correlations ranged from 0.831 to 0.890, and Spearman correlations from 0.812 to 0.883 (Table 3). The corrected Pearson and Spearman coefficients showed a consistent pattern. High correlations indicate substantial shared information among the three aggregate scores.

**Table 3:** Pairwise organization-level correlations.

Pair	Pearson r	Spearman rho
Supervision with Teaching & Learning	0.831	0.812
Supervision with Overall Experience	0.890	0.883
Teaching & Learning with Overall Experience	0.857	0.836

*N* = 743 organizations for all pairs. Coefficients describe between-organization associations from the same survey source.

Supervision was positively associated with Overall Experience (c = 0.838) and Teaching & Learning (a = 0.789). In the outcome equation, Teaching & Learning was positively associated with Overall Experience conditional on Supervision (b = 0.375), while Supervision retained a positive conditional association (c′ = 0.543). All four coefficients had *p* < 0.001 (Table 4). The outcome model had conventional R^2^ = 0.8367 and adjusted R^2^ = 0.8362. These values summarize in-sample variation in the aggregate Overall Experience score.

**Table 4:** Primary linear regression paths.

Path	B	HC3 SE	Beta	95% CI	R^2^/adjusted R^2^
c: X with Y	0.838	0.023	0.890	0.794 to 0.883	0.7925/0.7923
a: X with M	0.789	0.024	0.831	0.742 to 0.835	0.6904/0.6900
c′: X with Y, given M	0.543	0.032	0.577	0.480 to 0.606	0.8367/0.8362
b: M with Y, given X	0.375	0.035	0.377	0.306 to 0.443	0.8367/0.8362

*N* = 743. X, Supervision; M, Teaching & Learning; Y, Overall Experience. B is unstandardized; beta uses organization-level standard deviations. All four path *p* values are <0.001. Confidence intervals use HC3 standard errors and residual t degrees of freedom. R^2^ values in the last two rows refer to the same two-predictor outcome model.

### 3.3 Indirect association and magnitude

The linear product ab was 0.295 (bootstrap 95% CI 0.240–0.351). The standardized product was 0.314 (95% CI 0.258–0.367). Under this statistical decomposition, a 10-point between-organization difference in Supervision corresponds to 2.95 points of the fitted Overall Experience difference in the indirect component (95% CI 2.40–3.51). This contrast compares observed organizations under the specified regression model. Figure 1 reports the unstandardized and standardized paths. The result indicates a positive indirect association in the cross-sectional decomposition.

**Figure 1:**
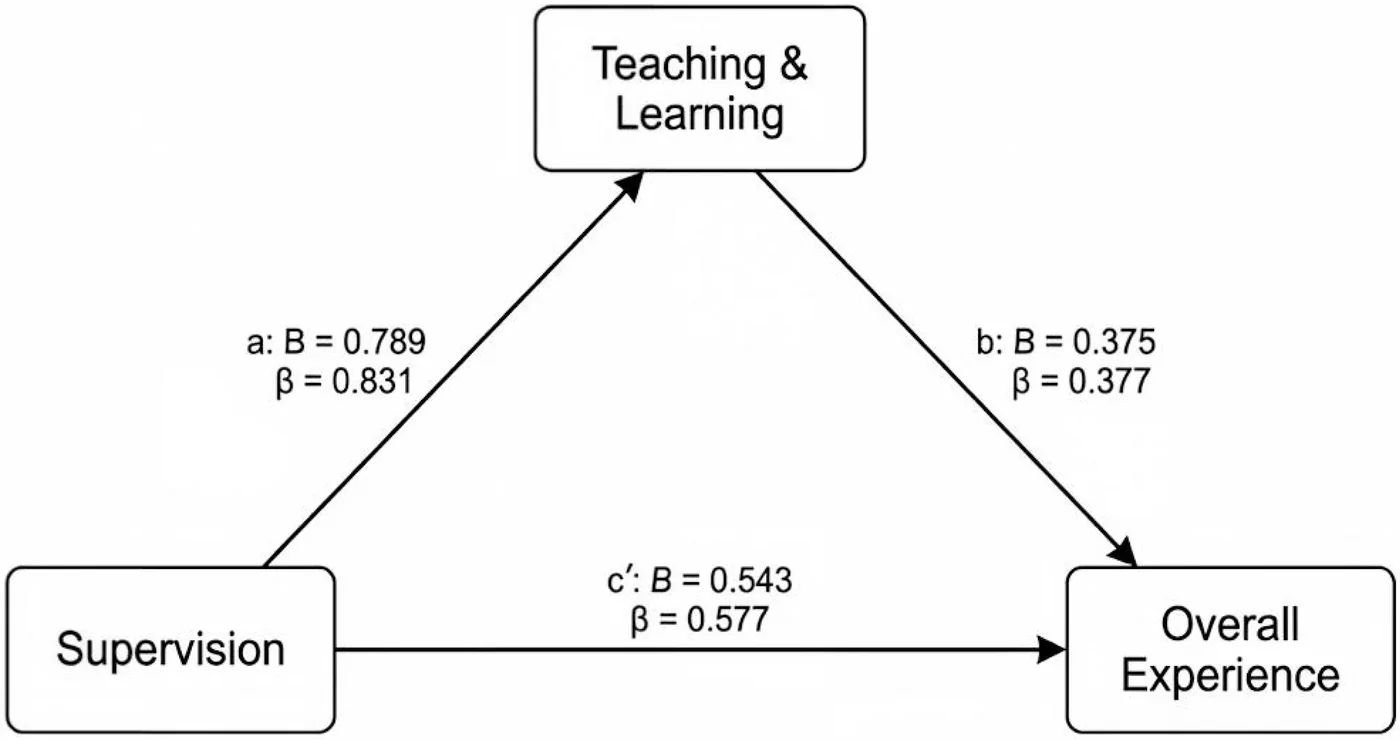
Organization-level regression decomposition. Arrows specify the ordering of a cross-sectional organization-level regression decomposition. B denotes unstandardized coefficients and beta denotes standardized coefficients. Indirect association ab = 0.295 (95% bootstrap CI 0.240–0.351); standardized indirect association = 0.314 (95% bootstrap CI 0.258–0.367). Path intervals and HC3 standard errors appear in Table 4. The indirect intervals use 10,000 organization-level percentile bootstrap resamples. *N* = 743.

### 3.4 Diagnostics and sensitivity

Scatterplots and residual plots suggested some curvature at the lower end of the score distribution, and normal quantile plots showed residual tail departures (Supplementary Figures S1, S2). The quadratic X term in the intermediate-variable model was supported by an exploratory robust Wald test (F = 8.65, *p* = 0.0034), whereas the joint outcome-model quadratic terms were not (F = 1.64, *p* = 0.195). The linear decomposition should therefore be read as an overall summary. Quadratic intermediate-model contrasts for a 10-point increase from the 25th, 50th, and 75th Supervision percentiles yielded indirect components of 2.94, 2.79, and 2.59 points, respectively; their bootstrap intervals excluded zero (Supplementary Table S7).

Removing the 24 boundary-score organizations retained 719 organizations and produced ab = 0.300 (95% CI 0.245–0.355). Excluding 58 influential organizations retained 685 and produced ab = 0.248 (95% CI 0.209–0.289). Adjustment for Induction, Facilities, and Workload yielded ab = 0.124 (95% CI 0.087–0.163). Thus, the positive direction persisted, while the magnitude depended on the specification, particularly adjustment for other environmental indicators.

The count-weighted estimate was 0.323 (95% CI 0.269–0.378). Restriction to the 288 organizations with displayed counts of at least 10 yielded 0.256 (95% CI 0.164–0.351). The correctly decoded original 500 organizations yielded 0.306 (95% CI 0.231–0.381), placing the value correction alongside the recovery of additional organizations in the reporting trail. In the ICB subset, the cluster-bootstrap estimate was 0.287 (95% CI 0.230–0.346). Table 5 and the Supplementary Tables provide the complete reporting trail. These findings show persistence of a positive association across the examined specifications.

**Table 5:** Indirect associations across analysis specifications.

Analysis	N	ab	95% bootstrap CI
Primary unweighted	743	0.295	0.240 to 0.351
Exclude boundary scores	719	0.300	0.245 to 0.355
Exclude influential cases	685	0.248	0.209 to 0.289
Adjust additional indicators	743	0.124	0.087 to 0.163
Corrected original organizations	500	0.306	0.231 to 0.381
Weight by displayed counts	743	0.323	0.269 to 0.378
Displayed counts ≥10	288	0.256	0.164 to 0.351
ICB subset, cluster bootstrap	676	0.287	0.230 to 0.346

Each interval uses 10,000 percentile bootstrap resamples. Organizations are sampled for the first seven analyses; 42 ICBs are sampled for the final analysis. Boundary exclusion removes 24 organizations; Cook's-distance exclusion removes 58 using 4/743. The covariates are Induction, Facilities, and Workload. Count weights give greater emphasis to larger reporting groups. Full paths and standardized products are in Supplementary Tables S4A–S4G. X, Supervision; M, Teaching & Learning; Y, Overall Experience; ab is the product of the X–M and conditional M–Y coefficients. ICB, integrated care board.

## 4 Discussion

### 4.1 Principal findings

Across 743 NETS reporting organizations, Supervision, Teaching & Learning, and Overall Experience showed strong positive associations. The outcome model combined a positive association for Supervision with a further positive association for Teaching & Learning. The estimated indirect component was 0.295, with a standardized value of 0.314. Its direction persisted across the examined analytical specifications, while its magnitude varied, particularly after inclusion of other placement-environment indicators. Taken together, these findings show that favorable ratings of supervision and learning opportunities form a closely connected pattern within organization-level placement appraisal.

The practical size of the indirect component is interpretable on the published score scale. A 10-point between-organization contrast in Supervision corresponded to a fitted indirect component of 2.95 Overall Experience points under the linear model. The remaining conditional Supervision coefficient indicates that the relationship with overall experience also includes a component associated with supervision beyond the measured opportunities indicator. Educationally, this pattern draws attention to both the relational quality of guidance and the organization of learning activity. It provides a basis for reviewing these dimensions together when discussing survey findings with placement teams.

The extraction audit was consequential for the empirical findings. Correct decoding reconciled all 6,500 cells in the archived matrix with the independently retrieved flat table, and complete retrieval increased the available organization set from 500 to 743. The primary scores were complete across this set. The analysis restricted to the corrected original 500 organizations produced an indirect estimate of 0.306, close to the estimate in the complete retrieved sample. This comparison places the contribution of corrected values alongside the contribution of broader organization coverage. The reporting trail in Supplementary Table S1 allows these changes to be followed directly from data preparation to the revised analysis.

### 4.2 What the scores say about placement experience

The score distributions indicate generally favorable reported experiences among the observed organizations. Mean scores were 77.84 for Supervision, 73.33 for Teaching & Learning, and 81.24 for Overall Experience, with corresponding medians in similar ranges. This positive overall pattern is consistent with accounts in which students value supportive staff and meaningful involvement in clinical work. Cant and colleagues found that Australian students’ narratives commonly described a positive learning culture and identified staff support as an organizing theme (8). In the English student-led placement evaluation, students described confidence, collaborative learning, and support from several members of the clinical team (9). The NETS pattern provides a complementary view of favorable experience at the reporting-organization level.

At the same time, the distribution across domains can help guide a more focused educational discussion. Teaching & Learning had the lowest mean of the three indicators. Its items concern access to opportunities, permission to attend activities, learning across professions, and simulation. These are concrete areas for reviewing how educational participation is arranged alongside clinical service. A useful local discussion would ask which opportunities are routinely accessible, which depend on the placement or shift, and how learners obtain permission and support to participate. The distinct item content of the indicators provides the basis for interpreting their relative profile.

Recent studies demonstrate the value of retaining this domain-level detail. Guejdad and colleagues found variation across leadership, supervision, and nurse-teacher dimensions in Morocco (10). Aldhahir reported differences between supervision and pedagogical-atmosphere ratings among respiratory therapy students (13). A survey of medical imaging students in Ghana combined generally positive experiences with concerns about supervision, crowding, and resources (35). Although the populations and instruments differ, these studies show that overall ratings can coexist with meaningful differences between domains and identify specific priorities for improvement. The same approach is useful when reviewing NETS results: the overall score provides context, while the component items direct attention to the practical arrangements learners encounter.

The strong joint fit of Supervision and Teaching & Learning with Overall Experience is also informative for survey interpretation. The two-predictor model summarized 83.7% of the observed aggregate outcome variation. This degree of covariance suggests that organizations’ ratings reflect a substantially shared pattern of perceived placement quality. Part of this covariance may reflect overlapping educational content and a common tendency to appraise the placement favorably or unfavorably. Thus, the high R2 does not by itself demonstrate that the indicators measure distinct constructs, and the indirect component may partly summarize shared appraisal rather than a separate educational process. The indicators are useful as an associated profile when identifying organizations for follow-up discussion. The remaining variation provides a reason to examine further dimensions, including induction, resources, workload, and the composition of learning opportunities. Qualitative feedback can help explain why organizations with similar component ratings receive different overall appraisals.

### 4.3 Comparison with supervision and workplace learning research

The present results connect with a literature in which support is repeatedly associated with the accessibility and perceived value of clinical learning. In Pront and colleagues’ review, learning-focused supervision involved competencies extending across educational activity and supervisory relationships (4). In the broader nursing literature, syntheses by Zonneveld and by Gill-Meeley describe a diverse set of supervision practices, including individual and group formats with different professional purposes (14, 15). NETS contributes a focused set of learner-facing indicators to this discussion: quality of supervision, timing and usefulness of feedback, and review of progress. Their association with learning opportunities suggests that these everyday supervisory practices are relevant to the broader organization of the placement experience.

Supervisors’ accounts help explain why these practices depend on local working conditions. Benny and colleagues’ qualitative study of mental health nurse preceptors identified time demands, additional workload, creating a safe environment, and the provision and receipt of feedback as prominent themes (36). Awiagah and colleagues likewise highlighted both individual and system-level conditions in the implementation of supervision (16). These findings place supervision within staffing, scheduling, and institutional relationships. The reduction in the NETS indirect component after adjustment for Induction, Facilities, and Workload is consistent with supervision and learning opportunities sharing information about this wider placement environment.

Feedback research offers a more specific interpretation of the relational dimension. The educational alliance foregrounds supervisors’ engagement with learners and the credibility of the relationship in which feedback is exchanged (24, 25). Recent work by Kearney and colleagues found that medical and pharmacy students’ experiences ranged from generic, overly favorable comments to a more confrontational feedback culture (37). These contrasting experiences show why the usefulness of feedback depends on its purpose, context, and interpersonal delivery. NETS questions about timely and constructive feedback capture an appraisal of this process, linking the supervision score to an aspect of learning that students can recognize in daily practice.

Examples from clinical education also connect feedback with the activities available to learners. Tran and colleagues describe changes to tasks in a primary care rotation intended to strengthen students’ use of feedback (38). Reierson and colleagues found that nursing students learning electronic documentation valued mentoring and feedback during placement, alongside prior academic preparation and peer learning (39). These studies give practical content to the association between Supervision and Teaching & Learning: feedback is embedded in opportunities to perform, reflect on, and revisit clinical tasks. The correspondence between the two NETS indicators can therefore be interpreted in relation to the joint organization of guidance and participation.

The pattern is also compatible with the affordances perspective. Billett's account connects opportunities offered by the workplace with learners’ engagement in those opportunities (22, 23). Inocian and colleagues reported relationships between clinical learning environment dimensions, including affordances and engagement, and aspects of nursing students’ caring behaviours (40). Hepburn and colleagues’ review of rural and remote placements emphasized interpersonal factors, learner engagement, and the supervisor's role in reported learning experiences (41). The NETS analysis extends the descriptive scope to a heterogeneous set of reporting organizations and estimates the joint pattern of supervision, opportunities, and overall appraisal within one public reporting system.

Related studies have examined other outcomes and levels of analysis. Mukhalalati and colleagues connected students’ environment perceptions with professional identity, while Karimi Mirzanezam and colleagues examined academic adjustment (11, 12). Savage and colleagues used path analysis to examine organizational support, psychological safety, stress, and burnout among residents across three institutions (42). Zhu and colleagues investigated relationships between clinical environment factors, resilience, and caring behaviours among nursing interns (43). These studies demonstrate the range of educational and professional outcomes associated with learning environments. The contribution of the present analysis is the organization-level decomposition of three specified NETS ratings, adding evidence about their joint use in placement evaluation.

### 4.4 Robustness and the organization of placement quality

The sensitivity analyses help characterize which features of the observed data contribute to the estimated association. Removing boundary-score organizations produced an indirect estimate of 0.300, close to the primary estimate. Excluding observations identified by Cook's distance reduced it to 0.248 while retaining a positive interval. The 24 boundary-score organizations and 58 influential organizations therefore affected the estimated magnitude differently. Reporting these analyses together provides a fuller account of stability across the score distribution than a single significance statement would provide.

Reporting size was another relevant feature. The public displayed counts ranged from 3 to 1,357, with a median of 6. The primary analysis describes the relationship across equally weighted organizations, while the count-weighted analysis gives greater emphasis to organizations with larger reporting groups. The positive indirect estimates under both approaches, together with the analysis restricted to counts of at least 10, show that the general pattern is visible under these different emphases. The count-weighted estimate of 0.323 and the minimum-count estimate of 0.256 also indicate that the magnitude responds to the definition of the analytical population and its weighting.

The additional-indicator model produced the largest attenuation, reducing the indirect component from 0.295 (95% CI 0.240–0.351) to 0.124 (95% CI 0.087–0.163) after simultaneous adjustment for Induction, Facilities, and Workload. This attenuation is consistent with the unadjusted association sharing substantial information with the wider placement environment. Conceptual frameworks of the clinical learning environment describe interactions between organizational arrangements, social relationships, and material conditions (5–7). For example, workload pressures may limit time for supervisory contact and attendance at learning activities, while induction and facilities may be relevant to access to those activities. These are plausible contextual interpretations of the organization-level pattern; the present analysis did not test these processes within individual learners.

Because the three contextual indicators entered the models jointly, this comparison cannot identify how much of the attenuation is attributable to Workload alone or establish that it is more important than Induction, Facilities, or Supervision. The reduction in the coefficient product is not a proportion of outcome variance explained by context. It may reflect shared environmental conditions, overlapping appraisal, or adjustment for closely connected dimensions of the same placement experience. The remaining positive indirect component describes a conditional association between organizations. These data cannot distinguish contextual confounding from shared measurement or overadjustment, so neither the primary nor the adjusted estimate establishes an independent causal pathway.

The clustering and functional-form analyses add further detail. The ICB-level bootstrap retained a positive indirect estimate in the subset of 676 organizations with an unambiguous assignment. This analysis reflects one identifiable administrative grouping within the reporting system. The quadratic intermediate-model contrasts varied from 2.94 to 2.59 points across the selected starting percentiles, suggesting some variation in the fitted indirect component across the score range. These results support a summary that retains both the consistency of the positive pattern and the observed variation in its magnitude across models and subsets.

### 4.5 Implications for educational quality and further research

For education leads, the findings support reviewing Supervision, Teaching & Learning, and Overall Experience together. An organization-level profile can initiate a structured discussion with clinical educators, placement managers, and learners. It can identify organizations for local inquiry, but cannot show that an individual learner with better supervision necessarily has better learning opportunities or a better placement experience. A practical starting point is to compare the broad pattern across the three domains and then return to the item content. Questions about timely feedback, review of progress, attendance at learning activities, and access to simulation offer a more precise agenda than an overall score alone. The review can then connect each item with the people and working arrangements responsible for delivering that part of the placement.

Continuity and preparation are useful areas for local improvement work. Learners need a clear route to supervision, an understanding of what they can participate in, and opportunities to discuss progress across the placement. The findings of Guejdad and colleagues regarding supervisor meetings, together with the preparation issues identified in the English student-led placement evaluation, provide empirical reasons to examine these arrangements (9, 10). A placement team could review how learners are introduced to the service, how supervisory contact is maintained, and how expectations are revisited when responsibilities change. Local evaluation can record whether these arrangements were actually delivered and how learners experienced them.

Feedback development offers a second area for educational action. The studies by Tran and Kearney suggest that feedback requires both usable activities and a shared understanding of its purpose (37, 38). Educational teams can examine whether feedback is connected to an observed task, whether learners have an opportunity to discuss it, and whether a subsequent activity allows them to act on it. Faculty development can incorporate the learner's perspective on credibility and support within the educational alliance (25, 26). Evaluation would benefit from considering the availability, usefulness, and uptake of feedback as related features of practice.

Support for participation also warrants attention. Psychological safety provides a framework for considering how learners ask questions, disclose uncertainty, and seek help (27, 28). Howard and Peirson's study of group supervision highlights the relevance of confidence and trust to engagement, including in online formats (29). Intervention evidence offers further guidance: Ko and colleagues’ systematic review and meta-analysis found reductions in placement-related stress and anxiety following pre-clinical interventions, although the approaches and outcomes varied (44). Together, these sources support locally evaluated preparation and support strategies alongside changes to clinical learning opportunities.

The findings further encourage coordination between education providers and clinical services. An educationally useful opportunity depends on access to activities, staff availability, physical or digital resources, and a learner's ability to participate. Evidence from electronic documentation learning and rural placements illustrates how these conditions vary with tasks and settings (39, 41). The vision of exemplary learning environments proposed by van Schaik and colleagues places collaboration and continuous improvement at the centre of the relationship between education and clinical work (45). Using NETS feedback within a shared review process can help different stakeholders identify where their responsibilities intersect.

Further research can connect this organization-level description with more detailed accounts of educational practice. Mixed-methods studies could purposively sample organizations with contrasting indicator profiles and examine how supervisory contact, access to activities, and workload are organized. Linking repeated placement evaluations with administrative and educational information would allow researchers to study changes over time. Individual-level data could connect reported opportunity and support with engagement, professional identity, and assessed learning. Measurement work could examine the structure of the NETS indicators using item responses and compare their performance across professions and training stages, building on established and recent clinical-environment measurement research (17–20).

### 4.6 Limitations

The study is ecological and cross-sectional. Its coefficients describe differences among public reporting organizations and cannot establish temporal mediation, individual learning gains, or the effects of changing supervision. Between-organization associations need not have the same direction or magnitude as associations among learners within an organization. Aggregation also obscures variation between placements and learner groups within each reporting organization. The three indicators share a survey source and overlap in their educational content. Common evaluative tendencies, unmeasured provider characteristics, and reverse or reciprocal relationships may contribute to their covariance. The published aggregates do not permit item-level reliability analysis, discriminant-validity assessment, or adjustment for learner characteristics. Thus, the statistical indirect component should not be equated with a demonstrated learning mechanism (46).

The reporting units are heterogeneous and include trusts, primary-care organizations, and other providers. Reporting-view filters, participation, and public masking limit representativeness; a completed query does not establish coverage of the whole target workforce. Displayed counts measure reporting size but do not provide every item's effective denominator or sampling variance. Some administrative mappings are multiple or missing, and the ICB analysis cannot account for dependence through other placement networks or repeated learner participation. Cross-study comparisons also involve different professions, instruments, and units of analysis, which limits direct comparison of coefficients and score levels.

The linear models summarize bounded scores with residual tail departures and some curvature. HC3 standard errors and bootstrap intervals do not correct misspecified functional form or unmeasured confounding. The covariate specification was retained from the original analysis on grounds of availability and conceptual relevance; its selection timing cannot be established from the available records. It is therefore exploratory, and adjustment may remove shared environmental variance or introduce overadjustment. The sensitivity results characterize variation across the examined specifications rather than identifying a sufficient causal adjustment set. Finally, the educational actions discussed above draw on the wider literature and require local evaluation; their effectiveness was not assessed by this NETS analysis.

## 5 Conclusions

Clinical supervision, perceived teaching and learning quality, and overall placement experience were strongly associated across 743 NETS 2025 reporting organizations. Teaching & Learning retained a positive association with Overall Experience conditional on Supervision, and the regression decomposition identified a positive indirect component across several analytical specifications. The magnitude varied with additional placement indicators, reporting-size emphasis, and sample definition. These findings support interpreting the NETS indicators as a connected profile of placement experience and using their item content to guide discussions of supervision, feedback, and access to learning opportunities. The estimates describe differences between organizations and should not be extrapolated to individual learners or interpreted as effects of changing supervision. The analysis provides an empirical basis for focused organizational review and for further longitudinal and mixed-methods research on how educational participation is organized in clinical settings.

## Data Availability

Publicly available datasets were analyzed in this study. This data can be found here: NHS England National Education and Training Survey 2025 reporting tool: https://app.powerbi.com/view?r=eyJrIjoiMWZmYjNiYTQtNTQ2OS00YzlhLWIxYWEtOWVkYmY4ZTM3MTM2IiwidCI6IjM3YzM1NGIyLTg1YjAtNDdmNS1iMjIyLTA3YjQ4ZDc3NGVlMyJ9.
