# Supplementary material for: Teaching and learning in clinical placements: associations of supervision and learning opportunities with placement experience in NETS 2025
Source: Front Med (Lausanne). 2026 Oct 28;13:1947428. doi: 10.3389/fmed.2026.1947428 (PMC13751437; doi:10.3389/fmed.2026.1947428)
Supplement: Supplementary file 1 [file Datasheet1.pdf]

Supplementary methods and results

S1. Extraction audit and reproducibility

The retained public Power BI matrix response used a primary-window request of 5,000 and a secondary top count of 200. The server returned a reduction-limit warning, a false completeness flag (IC = false), 500 organization rows, and a continuation token ending at Royal Cornwall Hospitals NHS Trust. The client request size therefore did not establish complete retrieval. The corrected flat query returned IC = true, 743 organizations, and no continuation token or warning.

Matrix cells use a repeated-value bitmask R and a null-value bitmask Ø. Values absent from the C array cannot be assumed missing: a repeated bit carries forward the prior value. A shade/category field can also occupy the remaining C slot when the score itself is repeated. The corrected decoder follows these masks across cells and organization boundaries. All 6,500 cells from the retained matrix match the independent flat query after correct decoding. The original CSV has 426 discordant cells, including artificial missing values and numbers derived from non-score fields.

Table S1 summarizes the extraction discrepancies and their verified corrections.

Table S1 Examples and consequences of extraction correction

Item	Original file or report	Verified correction
Available organizations	500 rows, ending alphabetically at Royal Cornwall	743 organizations in completed query
Complete primary-indicator records	472 after 28 apparent exclusions	743; zero missing on each primary indicator
3Sixty Healthcare Limited, Overall Experience	Missing	91.6667
Alban Healthcare PCN, Facilities	0	62.5000
Andover PCN, Facilities	2	83.3333
Primary indirect association	0.1447 [0.0552, 0.2912]	0.2954 [0.2405, 0.3507]
Corrected original 500 organizations	Not available in original paper	0.3064 [0.2312, 0.3805]

Examples were checked against both the archived response and the complete flat query. Original estimates are reproduced only as an audit trail and are superseded. Bracketed values are 95% percentile bootstrap intervals.

The query preserved HEI_Exclude = false, exclusion of organization codes starting EE, and the exact named-organization exclusions in the archived payload. The complete exclusion list is retained in the authors' extraction records. No further profession or training-stage restriction was added. The report refresh date is 23 April 2026 in both archived and live metadata. Retrieval dates were 7 July 2026 (original) and 20 September 2026 (correction). Organization names are the pivot keys; no duplicate keys remain. Official score masking is retained, and respondent-level data are not queried.

S2. Indicator content and scoring

The mapped content of the three primary indicators is summarized in Table S2.

Table S2 Mapped question content

Indicator	2025 code	Content summary	Response family
Supervision	Q20.1	Perceived overall quality of supervision	Quality
Supervision	Q20.2	Feedback received at the time it is needed	Quality
Supervision	Q20.3	Feedback that helps improve performance	Quality
Supervision	Q20.5	Timely review of progress toward learning outcomes	Quality
Teaching & Learning	Q19.3	Breadth of learning opportunities relevant to training	Quality
Teaching & Learning	Q19.4	Permission to attend required learning activities	Quality
Teaching & Learning	Q19.5	Opportunities to learn from others and other professions	Quality
Teaching & Learning	Q22.5	Access to simulation or immersive learning	Frequency
Overall Experience	Q19.2	Perception that those in charge value education	Quality
Overall Experience	Q20.7	Overall educational experience of the placement or post	Quality
Overall Experience	Q22.7	Welcoming, supportive and friendly staff	Frequency
Overall Experience	Q31.1	Willingness to recommend the location for care	Recommendation
Overall Experience	Q31.2	Willingness to recommend the location for work or training	Recommendation

Summaries are paraphrases, not quotations. Codes and group assignments come from the current Question Mapping 25 entity, matched to Scoring Table 25. Some mapping column names retain 2024 labels. Quality responses span least to most favorable quality; frequency responses span never to always; recommendation responses span least to most likely. The five scored categories map to 0, 25, 50, 75, and 100. Non-applicable or other unscored responses have no numeric score. Tables S9A–S9C reproduce the official wording and response options from the archived mapping CSV. No item-level response records were downloaded.

The three mapped groups contain distinct question codes but overlapping educational content. For example, the Overall Experience items concerning supportive staff and the overall educational experience are conceptually close to supervision and access to learning opportunities. The overlap concerns the meaning of the items rather than reuse of the same mapped question. All analyses use the report's official masked aggregate score. Shared respondents, evaluative tendencies, and conceptual overlap may contribute to covariance and high model fit; these associations do not establish that the indicators measure distinct

constructs. The published aggregates do not permit item-level discriminant-validity assessment. See Sections 2.3, 4.2, and 4.6 of the main manuscript.

S3. Sample completeness and reporting size

Table S3 reports score completeness and the displayed respondent-count distribution.

Table S3 Primary score completeness and displayed respondent counts

Indicator	Observed organizations	Missing scores	Count min / median / max
Supervision	743	0	3 / 6 / 1357
Teaching & Learning	743	0	3 / 6 / 1357
Overall Experience	743	0	3 / 6 / 1357

Displayed public masked counts describe reporting size. All retrieved organizations have complete primary scores; the 28 exclusions in the original analysis reflected decoding errors. The observable sample follows public reporting and masking rules.

S4. Full sensitivity estimates

Tables S4A–S4G report the full focal paths and indirect associations for the seven organization-level specifications. These estimates compare reporting organizations and do not describe associations among learners within an organization.

Table S4A Primary paths (N = 743)

Path	B	HC3 SE	Beta	95% CI	p
c: X with Y	0.838	0.023	0.890	0.794 to 0.883	<0.001
a: X with M	0.789	0.024	0.831	0.742 to 0.835	<0.001
c′: X with Y given M	0.543	0.032	0.577	0.480 to 0.606	<0.001
b: M with Y given X	0.375	0.035	0.377	0.306 to 0.443	<0.001

Indirect association = 0.295 (95% CI 0.240 to 0.351); standardized product = 0.314 (95% CI 0.258 to 0.367). X, Supervision; M, Teaching & Learning; Y, Overall Experience. c is X with Y; a is X with M; c′ is X with Y conditional on M; b is M with Y conditional on X. B is unstandardized; SE is the HC3 robust standard error. Path intervals are HC3 t intervals; indirect intervals use 10,000 percentile organization bootstrap resamples. The additional-indicator specification includes Induction, Facilities, and Workload in every equation. Beta and standardized products use unweighted within-analysis organization standard deviations, including the count-weighted specification.

Table S4B Boundary excluded paths (N = 719)

Path	B	HC3 SE	Beta	95% CI	p
c: X with Y	0.839	0.025	0.879	0.789 to 0.889	<0.001
a: X with M	0.787	0.026	0.815	0.735 to 0.838	<0.001
c′: X with Y given M	0.539	0.033	0.565	0.474 to 0.604	<0.001
b: M with Y given X	0.381	0.035	0.385	0.312 to 0.450	<0.001

Indirect association = 0.300 (95% CI 0.245 to 0.355); standardized product = 0.314 (95% CI 0.260 to 0.367). X, Supervision; M, Teaching & Learning; Y, Overall Experience. c is X with Y; a is X with M; c′ is X with Y conditional on M; b is M with Y conditional on X. B is unstandardized; SE is the HC3 robust standard error. Path intervals are HC3 t intervals; indirect intervals use 10,000 percentile organization bootstrap resamples. The additional-indicator specification includes Induction, Facilities, and Workload in every equation. Beta and standardized products use unweighted within-analysis organization standard deviations, including the count-weighted specification.

Table S4C Influence excluded paths (N = 685)

Path	B	HC3 SE	Beta	95% CI	p
c: X with Y	0.832	0.013	0.917	0.806 to 0.859	<0.001
a: X with M	0.789	0.024	0.841	0.743 to 0.835	<0.001
c′: X with Y given M	0.584	0.023	0.644	0.540 to 0.629	<0.001
b: M with Y given X	0.314	0.025	0.325	0.265 to 0.363	<0.001

Indirect association = 0.248 (95% CI 0.209 to 0.289); standardized product = 0.273 (95% CI 0.231 to 0.316). X, Supervision; M, Teaching & Learning; Y, Overall Experience. c is X with Y; a is X with M; c′ is X with Y conditional on M; b is M with Y conditional on X. B is unstandardized; SE is the HC3 robust standard error. Path intervals are HC3 t intervals; indirect intervals use 10,000 percentile organization bootstrap resamples. The additional-indicator specification includes Induction, Facilities, and Workload in every equation. Beta and standardized products use unweighted within-analysis organization standard deviations, including the count-weighted specification.

Table S4D Additional indicators paths (N = 743)

Path	B	HC3 SE	Beta	95% CI	p
c: X with Y	0.545	0.033	0.578	0.479 to 0.610	<0.001
a: X with M	0.464	0.041	0.489	0.383 to 0.546	<0.001
c′: X with Y given M	0.421	0.034	0.447	0.353 to 0.488	<0.001
b: M with Y given X	0.266	0.038	0.268	0.192 to 0.341	<0.001

Indirect association = 0.124 (95% CI 0.087 to 0.163); standardized product = 0.131 (95% CI 0.093 to 0.173). X, Supervision; M, Teaching & Learning; Y, Overall Experience. c is X with Y; a is X with M; c′ is X with Y conditional on M; b is M with Y conditional on X. B is unstandardized; SE is the HC3 robust standard error. Path intervals are HC3 t intervals; indirect intervals use 10,000 percentile organization bootstrap resamples. Induction, Facilities, and Workload were entered jointly in every equation. Compared with the primary estimate in Table S4A, the smaller indirect association cannot be attributed to Workload alone or interpreted as a proportion of outcome variance explained by context. The estimate describes a conditional organization-level association; shared environmental information, conceptual overlap, and possible overadjustment are discussed in Section 4.4 of the main manuscript. Beta and standardized products use unweighted within-analysis organization standard deviations, including the count-weighted specification.

Table S4E Original 500 corrected paths (N = 500)

Path	B	HC3 SE	Beta	95% CI	p
c: X with Y	0.839	0.029	0.892	0.781 to 0.896	<0.001
a: X with M	0.800	0.028	0.848	0.745 to 0.854	<0.001
c′: X with Y given M	0.532	0.043	0.566	0.449 to 0.616	<0.001
b: M with Y given X	0.383	0.047	0.384	0.291 to 0.475	<0.001

Indirect association = 0.306 (95% CI 0.231 to 0.381); standardized product = 0.326 (95% CI 0.251 to 0.399). X, Supervision; M, Teaching & Learning; Y, Overall Experience. c is X with Y; a is X with M; c′ is X with Y conditional on M; b is M with Y conditional on X. B is unstandardized; SE is the HC3 robust standard error. Path intervals are HC3 t intervals; indirect intervals use 10,000 percentile organization bootstrap resamples. The additional-indicator specification includes Induction, Facilities, and Workload in every equation. Beta and standardized products use unweighted within-analysis organization standard deviations, including the count-weighted specification.

Table S4F Count weighted paths (N = 743)

Path	B	HC3 SE	Beta	95% CI	p
c: X with Y	0.885	0.020	0.939	0.845 to 0.924	<0.001
a: X with M	0.826	0.017	0.870	0.793 to 0.859	<0.001
c′: X with Y given M	0.562	0.030	0.597	0.502 to 0.621	<0.001
b: M with Y given X	0.391	0.032	0.394	0.328 to 0.453	<0.001

Indirect association = 0.323 (95% CI 0.269 to 0.378); standardized product = 0.343 (95% CI 0.286 to 0.401). X, Supervision; M, Teaching & Learning; Y, Overall Experience. c is X with Y; a is X with M; c′ is X with Y conditional on M; b is M with Y conditional on X. B is unstandardized; SE is the HC3 robust standard error. Path intervals are HC3 t intervals; indirect intervals use 10,000 percentile organization bootstrap resamples. The additional-indicator specification includes Induction, Facilities, and Workload in every equation. Beta and standardized products use unweighted within-analysis organization standard deviations, including the count-weighted specification.

Table S4G Count ≥10 paths (N = 288)

Path	B	HC3 SE	Beta	95% CI	p
c: X with Y	0.866	0.020	0.938	0.827 to 0.906	<0.001
a: X with M	0.865	0.027	0.892	0.812 to 0.918	<0.001
c′: X with Y given M	0.611	0.053	0.661	0.507 to 0.714	<0.001
b: M with Y given X	0.296	0.056	0.311	0.185 to 0.407	<0.001

Indirect association = 0.256 (95% CI 0.164 to 0.351); standardized product = 0.277 (95% CI 0.179 to 0.378). X, Supervision; M, Teaching & Learning; Y, Overall Experience. c is X with Y; a is X with M; c′ is X with Y conditional on M; b is M with Y conditional on X. B is unstandardized; SE is the HC3 robust standard error. Path intervals are HC3 t intervals; indirect intervals use 10,000 percentile organization bootstrap resamples. The additional-indicator specification includes Induction, Facilities, and Workload in every equation. Beta and standardized products use unweighted within-analysis organization standard deviations, including the count-weighted specification.

S5. Administrative clustering

Table S5 gives the path estimates and inference accounting for ICB clustering.

Table S5 CR2 cluster-robust path inference

Path	B	CR2 SE	95% CI	Satt. df	p
a: X with M	0.779	0.025	0.727 to 0.830	24.7	<0.001
c′: X with Y given M	0.557	0.033	0.489 to 0.624	29.1	<0.001
b: M with Y given X	0.369	0.035	0.298 to 0.440	29.0	<0.001
c: X with Y	0.844	0.029	0.785 to 0.903	24.7	<0.001

N = 676 organizations in 42 NHS ICB clusters. Whole-ICB bootstrap ab = 0.287 (95% CI 0.230 to 0.346), with 10,000 valid resamples. The subset excludes 42 multiple ICB mappings, 19 missing mappings, and 6 outside-England mappings. X, Supervision; M, Teaching & Learning; Y, Overall Experience. c is X with Y; a is X with M; c′ is X with Y given M; b is M with Y given X. B is unstandardized; SE is the CR2 cluster-robust standard error. ICB, integrated care board; Satt. df, Satterthwaite degrees of freedom. Path intervals use Satterthwaite degrees of freedom. This specification accounts for clustering within the identified ICB groups.

S6. Functional form and diagnostic checks

Exploratory quadratic-term tests are shown in Table S6, and fitted indirect components at selected Supervision percentiles are shown in Table S7. Figures S1 and S2 display the score relationships and regression diagnostics.

Table S6 Exploratory HC3 quadratic-term tests

Sample / model	F	df1 / df2	p
primary / M-model quadratic X	8.65	1 / 740	0.0034
primary / Outcome quadratic X and M	1.64	2 / 738	0.1949
boundary_excluded / M-model quadratic X	11.87	1 / 716	<0.001
boundary_excluded / Outcome quadratic X and M	1.64	2 / 714	0.1944
influence_excluded / M-model quadratic X	1.25	1 / 682	0.2644
influence_excluded / Outcome quadratic X and M	1.43	2 / 680	0.2400

X, Supervision; M, Teaching & Learning; Y, Overall Experience. df1 and df2 are numerator and denominator degrees of freedom. Wald F approximations use HC3 covariance and residual degrees of freedom. The intermediate-variable model (M ~ X) adds X^2; outcome equation jointly adds X^2 and M^2. These are exploratory functional-form checks. Raw case-level Cook's distances, leverage and studentized residuals are retained in the authors' analysis records.

Table S7 Descriptive indirect components allowing a quadratic X–M relationship

Supervision contrast	Indirect component	95% bootstrap CI
71.24 to 81.24	2.940	2.399 to 3.474
77.34 to 87.34	2.787	2.276 to 3.297
85.42 to 95.42	2.586	2.082 to 3.096

X, Supervision; M, Teaching & Learning; Y, Overall Experience. Contrasts start at the 25th, 50th and 75th percentiles of Supervision and increase by 10 score points. The fitted M difference from $M \sim X + X^2$ is multiplied by b from $Y \sim X + M$. Intervals use 10,000 organization bootstrap resamples. These descriptive contrasts summarize the fitted organization-level relationships under the specified equations.

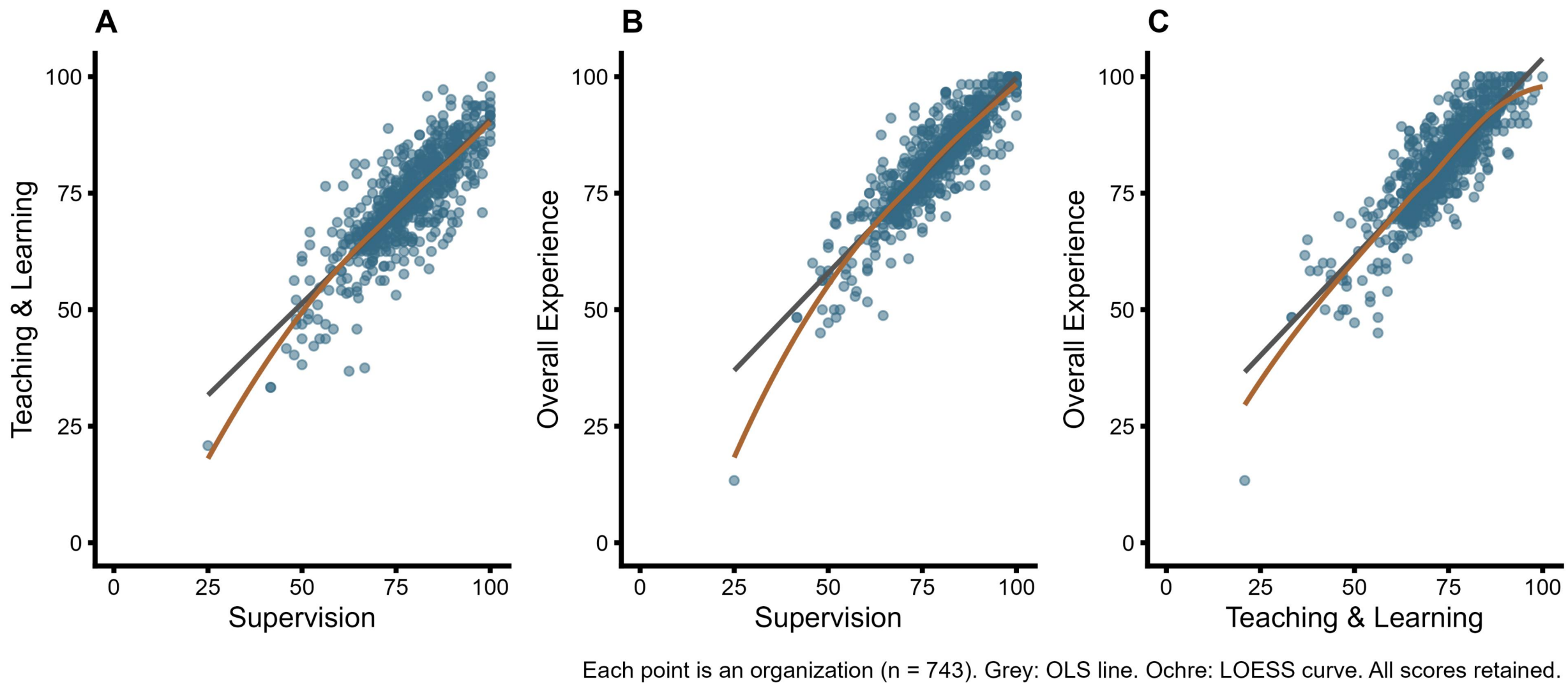

Figure S1 Pairwise organization-level score relationships. Each point represents one of 743 reporting organizations. Grey lines are ordinary least squares fits; ochre curves are locally estimated scatterplot smoothers. No cases have been removed for these plots. All axes use the normalized 0–100 score scale.

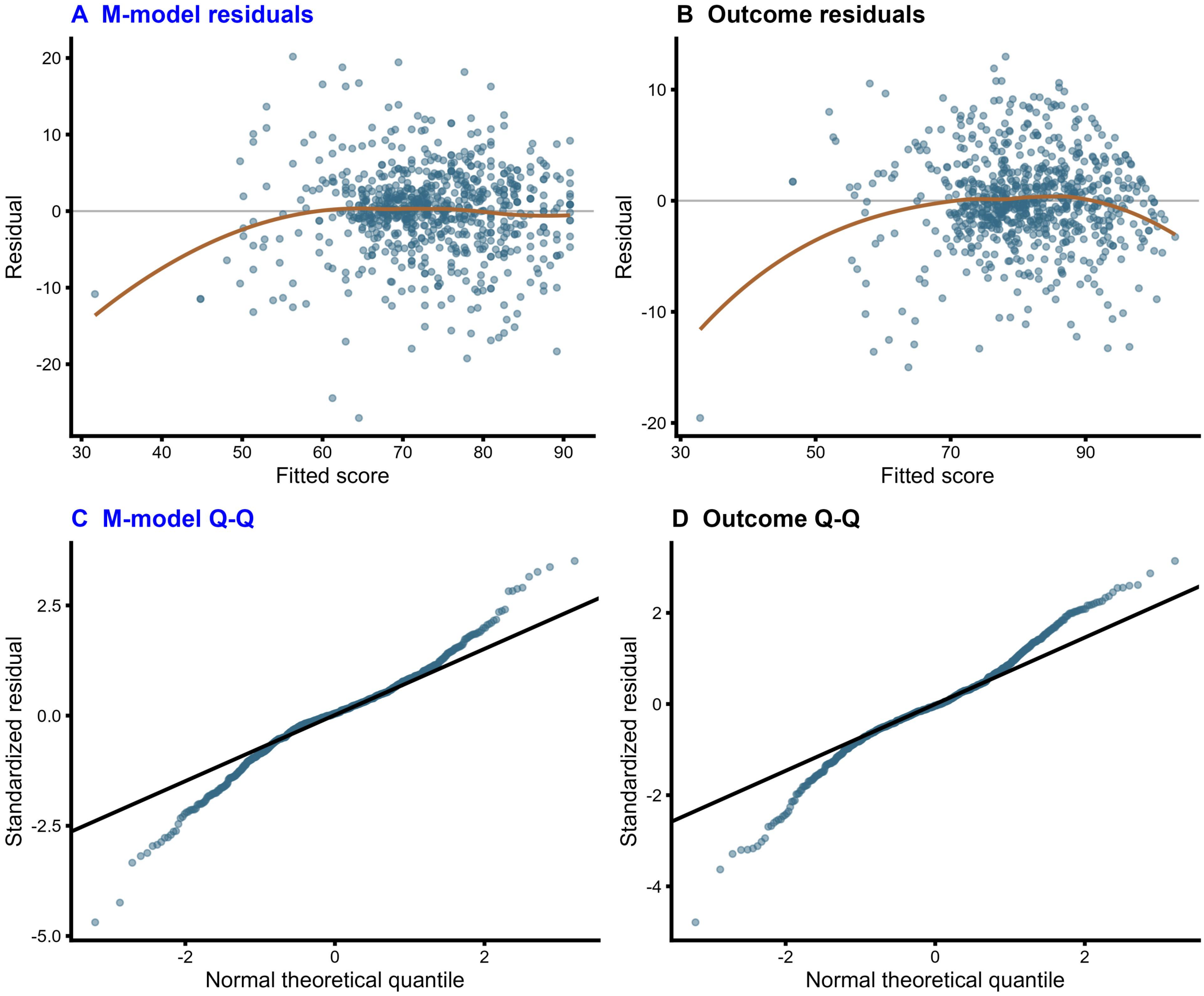

Figure S2 Primary regression diagnostics. X, Supervision; M, Teaching & Learning; Y, Overall Experience. Panels A and B show residuals versus fitted scores for the M ~ X and Y ~ X + M equations. In panels A and C, M-model denotes the intermediate-variable model (M ~ X), specifying a regression role without implying a causal mediator. Panels C and D show normal quantile plots of standardized residuals. The plots show some tail departures and curvature, complementing the quadratic-model sensitivity analysis. N = 743 in each model.

S7. Provider composition

Table S8 describes the public provider categories in the retrieved organization set.

Table S8 Available provider categories

Public provider label	Organizations
Care Home	9
Council Provider	4
Dental Practice	57
General Practice	287
Government	3
Hospice	3
Independent Provider	92
Multiple	42
Non-NHS Organisation	5
Optical Provider	2
Pathology Lab	3
Pharmacy Provider	35
Trust	178
Missing	23

A unique non-missing label is used when available. Multiple indicates more than one reported provider label. These categories describe reporting organizations. The summary assigns one reporting category per organization using its public administrative labels.

S8. Original question wording and response options

The following tables reproduce the source text in the archived NETS 2025 Question Mapping 25 metadata, retrieved on 20 September 2026. Table S2 contains our summaries; Table S9A contains the original mapped text, including its stems. Tables S9B and S9C list scored and unscored response options, respectively. Spelling and wording have been retained as recorded, including the phrase current or most practice placement in the Q22 stem. This is reporting metadata rather than a reconstruction of survey-screen routing. The normalized response scores were checked against Scoring Table 25. Source: NHS England NETS 2025 public reporting tool, cited in the main manuscript; the relevant question wording and response options are reproduced in Tables S9A-S9C. The processed analysis data and analysis code are available from the corresponding authors upon reasonable request.

Table S9A Original mapped questions

Indicator	Code	Original question text	Response family
Supervision	Q20.1	Please rate the quality of this practice placement or training post in the following areas: The overall supervision I received during the practice placement or training post	Quality
Supervision	Q20.2	Please rate the quality of this practice placement or training post in the following areas: Being given feedback on my performance at the time I needed it	Quality
Supervision	Q20.3	Please rate the quality of this practice placement or training post in the following areas: Being given feedback on my performance that was constructive (i.e. helped me to improve)	Quality
Supervision	Q20.5	Please rate the quality of this practice placement or training post in the following areas: Review of my progress towards my learning outcomes at the right time	Quality
Teaching & Learning	Q19.3	Please rate the quality of this practice placement or training post in the following areas: The range of learning opportunities to meet the needs of my training / programme	Quality
Teaching & Learning	Q19.4	Please rate the quality of this practice placement or training post in the following areas: Being allowed to attend learning opportunities to meet my curriculum or course requirements	Quality
Teaching & Learning	Q19.5	Please rate the quality of this practice placement or training post in the following areas: The opportunities to experience and learn from others, including those in other professions	Quality
Teaching & Learning	Q22.5	Please indicate how often you experienced or saw each of the following in your current or most practice placement / training post: I was able to access simulation and immersive learning opportunities	Frequency
Overall Experience	Q19.2	Please rate the quality of this practice placement or training post in the following areas: The extent to which I feel the people in charge value education and training	Quality
Overall Experience	Q20.7	Please rate the quality of this practice placement or training post in the following areas: The overall educational experience I gained in my current or most recent practice placement / training post	Quality
Overall Experience	Q22.7	Please indicate how often you experienced or saw each of the following in your current or most practice placement / training post: Staff were welcoming, supportive and friendly throughout the practice placement or training post	Frequency

Indicator	Code	Original question text	Response family
Overall Experience	Q31.1	Please think about your current or most recent experience and consider whether you would recommend the placement / training post for care and training: How likely are you to recommend this practice placement or training post location to friends and family if they ever need the care or treatment provided there?	Recommendation
Overall Experience	Q31.2	Please think about your current or most recent experience and consider whether you would recommend the placement / training post for care and training: How likely are you to recommend this practice placement or training post to friends and colleagues as a place to work or train?	Recommendation

Question wording is reproduced from the archived public mapping. Each question contributes to the indicated reporting group. Response families are decoding aids defined in Table S9B; unscored entries are reproduced in Table S9C. Distinct question identifiers do not exclude conceptual overlap between the indicators. The implications for interpreting their associations and the limits on measurement validation are discussed in Sections 2.3, 4.2, and 4.6 of the main manuscript.

Table S9B Scored response labels and normalized values

Normalized score	Quality	Frequency	Recommendation
0	Unsatisfactory	Never	Extremely unlikely
25	In need of improvement	Rarely	Unlikely
50	Satisfactory	Sometimes	Neither likely nor unlikely
75	Good	Often	Likely
100	Outstanding	Always	Extremely Likely

All three response families have five scored options. These values define the normalized rating scale. The analysis uses the published masked aggregate measure.

Table S9C Unscored entries in the mapping

Applicable question codes	Original unscored response entries
Q20.1, Q20.2, Q20.3, Q20.5, Q20.7	Not applicable to my experience or I haven't been in the placement long enough; I haven't been in the placement long enough; Not applicable or relevant to my experience
Q19.3, Q19.4, Q19.5, Q22.5, Q19.2, Q22.7	Not applicable to my experience or I haven't been in the placement long enough; I haven't been in the practice placement or training post long enough; Not applicable or relevant to my experience
Q31.1, Q31.2	Don't Know

Semicolon-separated alternatives are preserved as recorded in the mapping. These entries are unscored. The table documents the available question-mapping metadata; survey-screen routing and item-level counts were outside the public extract.
